# Construction of pH-sensitive targeted micelle system co-delivery with curcumin and dasatinib and evaluation of anti-liver cancer

**DOI:** 10.1080/10717544.2022.2048132

**Published:** 2022-03-09

**Authors:** Xiangle Zeng, Yawen Zhang, Xue Xu, Zhuo Chen, Lanlan Ma, Yushuai Wang, Xuliang Guo, Jianchun Li, Xiu Wang

**Affiliations:** School of Pharmacy, Bengbu Medical College, Bengbu, China

**Keywords:** pH response, targeted, hyaluronic acid, curcumin, dasatinib

## Abstract

Nanomedicine delivery systems can achieve precise drug delivery and reduce toxic side effects compared with traditional drug delivery methods, but further development is still needed to eliminate obstacles such as multiple drug co-delivery, uncontrolled drug-release, and drug-resistance. Herein, we designed a dual drug-loaded nanosystem (THCD-NPs) that selectively transports and targets tumor cells for the treatment of liver cancer. In this drug delivery system, hyaluronic acid (HA)-conjugated curcumin (Cur) and d-α-tocopherol acid polyethylene glycolsuccinate (TPGS) were used as selective drug-carrying vehicles to deliver dasatinib (DAS) to cancer cells for combined administration. The mean size of the nanoparticles was approximately 66.14 ± 4.02 nm with good *in vitro* stability. The nanoparticles were pH sensitive and could accelerate drug release at low pH conditions. *In vitro* experiments showed that THCD-NPs were significantly cytotoxic to HepG2 cells and could be effectively taken up by these cells. Detailed investigations also demonstrated its pro-apoptotic activity. *In vivo* NIR fluorescence imaging showed that the nanoparticles could accumulate efficiently at the tumor site. Meanwhile, *in vivo* experiments showed that THCD-NPs significantly inhibited tumor growth and reduced the toxic side effects of free drugs in a mouse solid tumor model. In short, the nanoparticles we prepared provide a new idea for the treatment of liver cancer.

## 1. Introduction

Liver cancer is one of the most common malignant tumors in the world (Anwanwan et al., 2020), and the incidence is increasing year by year. Currently, chemotherapy is the main treatment for liver cancer. However, small molecule chemotherapy drugs are usually injected into the body through intravenous injection, resulting in systemic effects. This approach is particularly beneficial in the treatment of metastatic cancer, but the attendant challenges include how to balance the therapeutic effect of drugs and how to reduce adverse side effects (Schirrmacher, 2019).

With the development of nanotechnology, the development of nano-sized drug carriers minimizes the side effects of small molecule chemotherapy drugs and solves other problems (Dai et al., 2019). For example, most chemotherapeutic drugs have poor water solubility, and their chemical structures are changed by covalently combining with hydrophilic drug carriers to increase their water solubility (Cheetham et al., 2017; Cabral et al., 2018; Shim et al., 2019; Wu et al., 2019). Nevertheless, these newly introduced parts or carriers need strict clinical trials (Zhao et al., 2016). In addition, the prepared nanoparticles can only accumulate in solid tumors by the EPR effect. The efficiency of passive targeting depends on the degree of tumor angiogenesis, the ability of cells to uptake drugs and the distribution of drugs in the tumor microcirculation, which limits the application of some carriers.

To develop nanoparticles with high targeting ability, researchers turned their target to active targeting preparations, and used the high affinity between targeted ligands and appropriate receptors overexpressed on the surface of target tumor cells to promote the effective internalization of tumor cells (Raj et al., 2021; Sun et al., 2021). Hyaluronic acid (HA) is a natural polysaccharide that is the main component of human somatic stroma, synovial fluid and other tissues. Therefore, nanoparticles with HA as carriers exhibit good biodegradability, biocompatibility, and non-immunogenicity (Pouyani & Prestwich, 1994; Li et al., 2013). At the same time, HA has high affinity with for the CD44 receptor on the cell membrane surface, and the expression of the CD44 protein in tumor cells is abnormally increased compared with that in normal human cells (Moriya et al., 2008; Idowu et al., 2012). Therefore, HA-modified nanoparticles may be preferentially taken up by tumor tissues through receptor-mediated endocytosis (Cheng et al., 2021).

In addition, a large number of tumor microenvironment-responsive nanoparticles were developed to further enhance the intracellular delivery of drugs and control the release of encapsulated drugs into tumor cells (Liu et al., 2014, 2016; Rao et al., 2014; Ye et al., 2016). Currently, the combination of active targeting of the micelle system and an intracellular pH-sensitive targeting strategy in cancer treatment is helpful to trigger the activation of nanosystems, thereby improving tumor targeting and intracellular drug delivery. This new approach provides an effective strategy to improve the therapeutic effect (Lai et al., 2021). Among them, ester bonds () are a kind of pH-triggered chemical structure, which is widely used to construct stimuli-responsive carriers (Wang et al., 2021; Zhang et al., 2021).

In recent years, owing to the unsatisfactory therapeutic effect of single chemotherapy drugs on tumors, combined medication has emerged on the stage of cancer treatment and has achieved surprising results. The synergistic effect of two or multiple medicines on tumors is much better than that of single drugs (Xiao et al., 2020; Yu et al., 2021). However, it is difficult to solve problems such as the superposition of multidrug adverse reactions, poor water solubility, and difficult co-delivery (Zhou et al., 2020). But, the emergence of drug-loaded nanoparticles has cleverly solved these problems. Dasatinib (DAS) is a multikinase inhibitor, which is used for chronic myelogenous leukemia with imatinib resistance or intolerance. DAS also shows good therapeutic effects on many solid tumors, including liver cancer, lung cancer, breast cancer, etc. (Mace et al., 2015; Yao et al., 2017; Zeng et al., 2018). It was found that DAS could inhibit cell cycle checkpoint kinase 1 (Chk1) and blocked HepG2 cells in G0/G1 (Corrales-Sanchez et al., 2020). Curcumin (Cur), a natural polyphenolic compound, has a variety of pharmacological effects (Adiwidjaja et al., 2017; Ciftci et al., 2018; Giordano & Tommonaro, 2019). It has been reported to block HepG2 cells at G2/M phase (Li et al., 2015). In the combination of DAS and Cur, DAS blocks tumor cells in G0/G1 phase, and Cur blocks tumor cells in G2/M phase. Both of them act on different cell cycles of tumor cells, and they are combined to exert anti-tumor effect. Nevertheless, there are many problems in practical applications. For example, DAS has a short half-life and is easily metabolized *in vivo* (Horinkova et al., 2019), and the poor solubility and stability of Cur severely limit the combination of the two drugs (Nelson et al., 2017). It is planned to use nanoparticles to solve the existing problems.

**Scheme 1.**
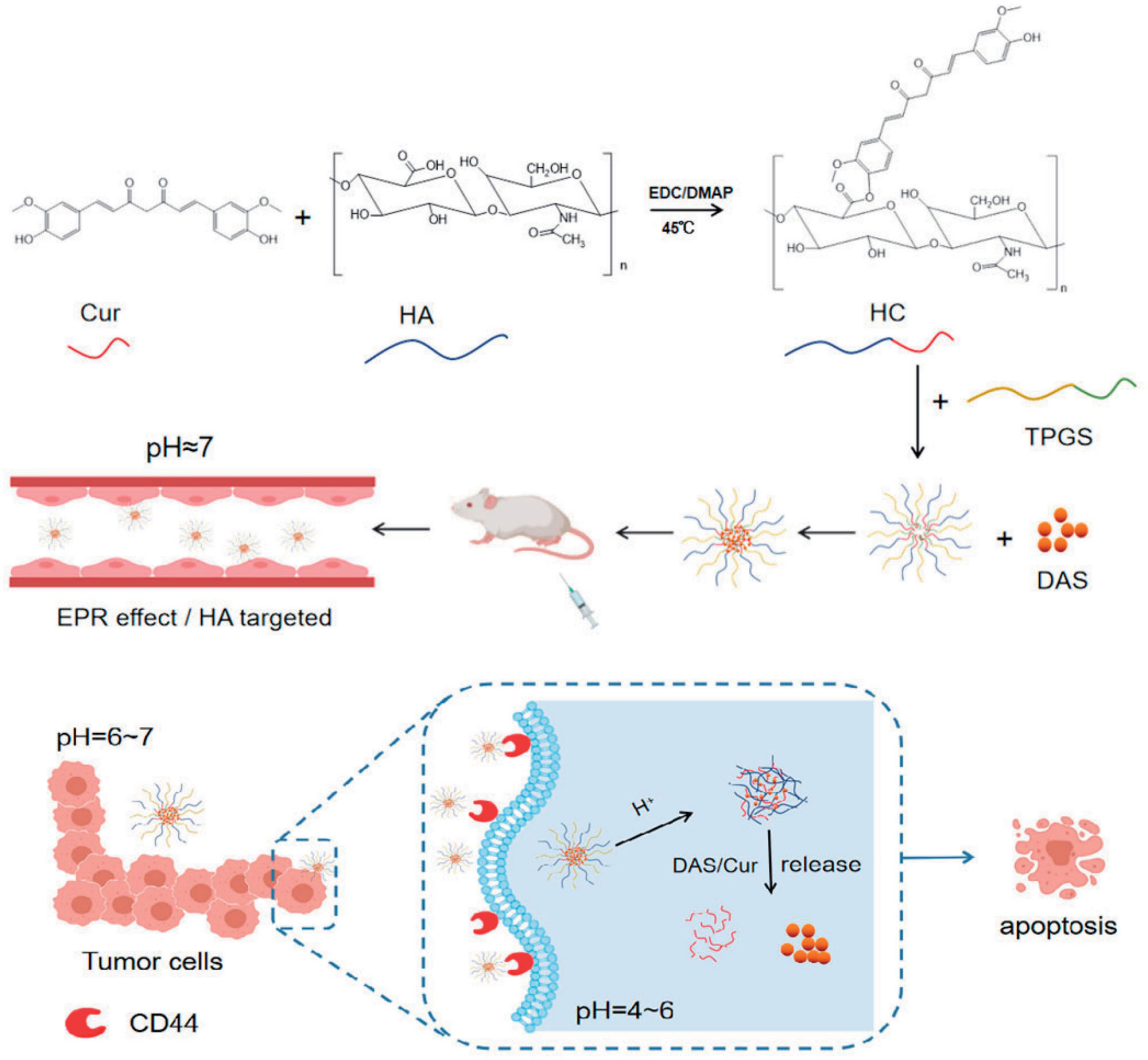
Proposed mechanism of HA targeting self-assembly nanoparticles (THCD-NPs) to co-delivery curcumin and dasatinib. The targeted dual drug-loaded nanoparticles have good stability under normal physiological environment and can be taken up by tumor cells overexpressing the CD44 protein after entering the systemic circulation. Meanwhile, the nanoparticles respond to the lower pH environment and then disintegrate and release the drug, and the two drugs act synergistically in different cell cycles to exert anti-tumor efficacy.

In this study, the hydrophobic drug Cur was connected with the natural polysaccharide HA by pH-sensitive ester bond to form the amphiphilic polymer HA-Cur. Then, the HA-Cur and d-α-tocopherol acid polyethylene glycolsuccinate (TPGS) were used as carriers, and DAS was used as the core to self-assemble into mixed micelle nanoparticles (THCD-NPs). Mixed micelles are used for co-delivery of DAS and Cur. Meanwhile, the solubility and stability of DAS and Cur can be well increased, and the retention time of drugs in the body can be prolonged. Among them, HA can actively target the overexpression of CD44 protein in tumor cells, and combined with the passive targeting of nanoparticles (EPR effect), the double targeting ensures that polymer micelles are taken up by tumor cells. pH-sensitive ester bonds can successfully activate the nanosystem and release drugs to inhibit tumor growth in the tumor micro-acid environment. TPGS can increase the stability of the micelles. In addition, THCD-NPs compensate for the lack of activity of the carrier and realize the synergistic effect of the carrier and the drug, which provides a new strategy for the treatment of liver cancer (Scheme 1).

## 2. Materials and methods

### 2.1. Materials

DAS, Cur, and TPGS were purchased from Macklin Biochemical Technology Co. Ltd. (Shanghai, China). HA was provided by Huaxi Biotechnology Co. Ltd. (Jinan, China, MW: 8814 Da). EDC and DMAP were from Aladdin Co. Ltd. (Shanghai, China). Dialysis bags with a molecular weight cutoff of 3.5 kDa (Viskase Companies, Inc., Darien, IL) were used. RPMI-1640, penicillin, streptomycin were obtained from Gibco (Carlsbad, CA). Fetal bovine serum (FBS) was purchased from Tian Hang Biotechnology Co. Ltd. (Hangzhou, China). DAPI staining reagent and phosphate-buffered saline (PBS) were purchased from Servicebio (Wuhan, China). Methylthiazolyldiphenyl-tetrazolium bromide (MTT) was purchased from Biofroxx (Einhausen, Germany).

### 2.2. Animals and cell culture

Human hepatocellular carcinoma cells HepG2 were obtained from EallBio Life Sciences Co. Ltd. (Beijing, China) and cultured in RPMI-1640 medium containing 10% FBS and 1% penicillin–streptomycin in an incubator at 37 °C, 5% CO_2_, and 90% relative humidity. The culture medium was changed approximately 2–3 days. BALB/C nude mice (male, 4–5 weeks, 18 ± 2 g) were purchased from Hunan Sja Laboratory Animal Co. Ltd. (Changsha, China). The animal experiments and feeding were carried out under SPF conditions, and the contents of animal experiments were in line with animal ethical standards.

The animal experimental procedures were approved by the Animal Health Committee of Bengbu Medical College on June 25 2021 (license no. 2021143), which met the requirements of animal ethics standards and the use Committee of Bengbu Medical College.

### 2.3. Synthesis and characterization of HA-Cur polymer

The HA-Cur polymer (HC) was synthesized according to the method described in the literature. Briefly, HA (0.1 mmol), DMAP (0.4 mmol), and EDC (0.4 mmol) were dissolved in 20 mL DMSO/H_2_O (v/v, 1:1), and the mixed solution was stirred at 60 °C for 1 h to activate carboxyl groups. Subsequently, Cur (0.4 mmol) was added to the reaction system to continue the reaction for 48 h at 45 °C. After the reaction, the product was added to a dialysis bag (MWCO 3.5 kDa) and dialyzed in deionized water for 48 h. After dialysis, the polymer HA-Cur was obtained by freeze-drying. The structure of HA-Cur was confirmed by UV-Vis spectrophotometer, DSC, and ^1^H NMR. As Cur is a hydrophobic drug, we also measured the saturation solubility of Cur and HC in water and PBS solutions respectively to examine the improvement of Cur's water solubility.

### 2.4. Preparation and characterization of mixed micelle nanoparticles (THCD-NPs)

In previous experiments, our group proved that the film dispersion method is the best way to prepare such micelles. Subsequently, the effects of the ratio of carriers on the micelle size and other indicators were investigated, and the effects of different ratios of dasatinib to carriers on the drug loading (DL) in the encapsulation efficiency (EE) were also investigated.

#### 2.4.1. Selection of different carrier ratio

The carriers were dissolved in 10 mL CH_3_OH/H_2_O mixed solvent (v/v, 1:1) with different proportions of HA-Cur/TPGS (3:1, 6:1, and 9:1, wt%), and the total amount of mixture was 20 mg. After ultrasonic treatment in ice bath for 20 min, the solvent was removed by a rotary evaporator, and the thin film was dissolved with appropriate amount of water. The no-load nanoparticles (THC-NPs) solution was obtained by 0.22 μm filter membrane filtration, and then the particle size of THC-NPs was measured.

#### 2.4.2. Selection of DAS dosage

After determining the optimal ratio of carriers, we screened the dosage of DAS. The carrier (HA-DAS/TPGS) materials with the optimal carrier ratio of 20 mg was dissolved in 10 mL CH_3_OH/H_2_O mixed solvent (v/v, 1:1), and then different doses of DAS were added (the mass ratios of DAS to carriers were 1:25, 1:20, 1:15, 1:10, and 1:5). The preparation method was the same as that described in Section 2.4.1. The ester bond structure was destroyed by strong alkali hydrolysis, and the concentration was measured by high-performance liquid chromatography (HPLC). The DL and EE were calculated by following equations:

(1) $$\text{DL}\%=\frac{\text{weight}\;\text{of}\;\text{drug}\;\text{in}\;\text{mixed}\;\text{micelles}}{\text{weight}\;\text{of}\;\text{mixed}\;\text{micelles}}\times 100\%\;$$

(2) $$\text{EE}\%=\frac{\text{weight}\;\text{of}\;\text{drug}\;\text{in}\;\text{mixed}\;\text{micelles}\;}{\text{weight}\;\text{of}\;\text{drug}\;\text{added}}\times 100\%\;$$

### 2.5. Characterization of the stability of THCD-NPs

THCD-NPs were incubated with PBS containing 10% FBS at 4 °C and sampled at set time points (0, 1, 3, 5, and 7 d). The size distribution was measured by Malvern particle size analyzer to reflect the stability of nanoparticles. Moreover, the dilution stability of THCD NPs with different dilution ratios (0, 100, 200, 400, 600, 800, and 1000) was also investigated.

### 2.6. pH-responsive release behaviors of DAS and Cur *in vitro*

DAS and Cur release in THCD-NPs was measured by dialysis at room temperature using PBS (pH 7.4, 6.0, and 5.0) containing 1% (w/v) Twen-80 at different pH values to simulate the normal physiological environment, tumor microenvironment, and lysosomal environment, respectively. Briefly, 4 mL of THCD-NPs solution was added to a dialysis bag (MWCO 3.5 kDa), and then immersed in release media with different pH values. At the preset times (0, 0.5, 1, 2, 4, 6, 8, 10, 12, 24, 36, and 48 h), 1 mL of release solution was taken and the same volume of release medium was supplemented. The released solution was filtered through a 0.22 µm microporous membrane, and then the concentration and the release degree of DAS and Cur in the released solution were calculated by HPLC.

### 2.7. The cell uptake of THCD-NPs

As Cur itself has fluorescence, the uptake behavior of nanoparticles by tumor cells was reflected by observing the fluorescence of Cur. Briefly, HepG2 cells, which grew well, were cultured overnight in 24-well plates at a density of 1 × 10^4^ cells per well and divided into four groups (HA + THCD-NPs, TPGS-Cur, HA-Cur, THCD-NPs). HA + THCD-NPs group was pre-incubated with 1 mg/mL HA for 0.5 h. Then, the THCD-NPs solution was incubated with the two groups of cells at 37 °C for 4 h.

The qualitative evaluation of cellular uptake was evaluated by fluorescence microscope. Briefly, after incubation, the cells were washed three times with PBS solution to remove uningested nanoparticles. Subsequently, they were stained with DAPI for 10 min at room temperature, washed three times with PBS solution, and immediately observed with a fluorescence microscope.

The quantitative evaluation of cellular uptake was evaluated by flow cytometry analysis. Briefly, after incubation, the medium was removed, and the cells were washed with PBS for three times. Cell suspensions were collected, and internalization was quantified by flow cytometry.

### 2.8. Anti-tumor effect of the nanomedicines *in vitro*

In this study, the MTT method was used to investigate the toxicity of THCD-NPs on HepG2 cells. In short, logarithmic growth HepG2 cells were seeded in 96-well plates at 5 × 10^3^ cells per well for 24 h. Subsequently, the old medium was replaced with 0.1 mL drug-containing medium, which contained different concentrations of DAS, Cur, DAS + Cur, and THCD-NPs (DAS to Cur molar ratio was fixed at 3:1). After incubation in the incubator for 24 or 48 h, 20 µL MTT was added to each well and incubated for another 4 h. After incubation, the absorbance at 490 nm was measured with a microplate reader, and the cell survival rate was calculated according to the following equation:

(3) $$\text{Cell}\;\text{viability}\;\%=\frac{A_{\text{measurement}}-A_{\text{blank}}}{A_{\text{control}}-{\;-A}_{\text{blank}}}\times 100\%\;$$

To further demonstrate the highly effective *in vitro* cytotoxicity of the co-doped NPs, the viability of the HepG2 cells was measured by co-staining with calcein acetoxymethylester (calcine AM) and propidium iodide (PI) to differentiate live and dead cells.

### 2.9. Apoptosis and cell cycle assessed by flow cytometry

The number of apoptotic cells was quantified using a kit containing annexin V-fluorescein isothiocyanate (V-FITC) and PI. First, HepG2 cells were inoculated at 4 × 10^5^ cells per well in six-well plates overnight and then incubated with fresh medium containing Cur, DAS, Cur + DAS, and THCD-NPs for 24 hours. Subsequently, the cells were digested, and apoptotic cells and living cells were collected by centrifugation (1000 rpm, 5 min). The cells were re-dispersed in the buffer solution, and V-FITC and PI staining were added according to the kit steps. The number of apoptotic cells was measured by flow cytometry. Living cells, early apoptotic cells, late apoptotic cells, and necrotic cells were named *Q*_1_, *Q*_2_, *Q*_3_, and *Q*_4_, respectively.

To analyze the cell cycle, the cells were washed after incubation with drugs (Cur, DAS, Cur + DAS, THCD-NPs) and collected by centrifugation (1000 rpm, 5 min). The cells were fixed in 75% ethanol overnight and stained with PI. Subsequently, the cell cycle was detected by flow cytometry.

### 2.10. Hemolysis test

The effect of THCD-NPs on red blood cells was investigated. In short, THCD-NPs at different concentrations were mixed equally with 2% red blood cell diluent. After incubation at 37 °C for 2 h, the mixture was centrifuged at 5000 rpm for 5 min. After centrifugation, the supernatant was collected to determine the absorbance at 570 nm. The relative hemolysis rate was calculated according to the following equation:

(4) $$\text{Relative}\;\text{hemolysis}\;\text{rate}\;\%=\frac{A_{\text{sample}}-A_{\text{PBS}}}{A_{\text{water}}-A_{\text{PBS}}}\times 100\%\;$$

### 2.11. Distribution of nanoparticles *in vivo*

To investigate the *in vivo* targeting of nanomedicine, tumor-bearing mice were divided into two groups (*n* = 3). Mice were intravenously injected with Cy5 and Cy5-labeled THC-NPs (the dose of Cy5 was 10 μg/mouse). After administration for 0 h, 1 h, 4 h, 6 h, 10 h, and 24 h, the tumor-bearing mice were anesthetized and placed in a small animal *in vivo* imager for fluorescence scanning and photographing.

### 2.12. *In vivo* antitumor and safety evaluation

BALB/C nude mice of HepG2 xenograft tumors were randomly divided into five groups (PBS group, DAS group, Cur group, DAS + Cur group, and THCD-NPs group). When the tumor volume reached approximately 100 mm^3^, nude mice were intravenously injected with different preparations (5 mg/kg) every three days for a total of six injections. The first day of treatment was defined as day 0. The body weight and tumor size of the mice were recorded every three days. Tumor volume was calculated according to the following equation:

(5) $$\text{Tumor}\;\text{volume}\;(V)=\frac{a\times b^{2}}{2}\;$$

where *a* is the tumor length and *b* is the tumor width.

After treatment, the mice were euthanized, and the tumor and major organs were completely stripped and fixed in 4% paraformaldehyde. Then, the tissues and organs were stained with Ki67 and H&E staining, and pathological analysis was performed under the microscope. Serum was collected for the detection of aspartate aminotransferase (AST), alanine aminotransferase (ALT), blood urea nitrogen (BUN) and creatinine (Cre) to reflect the safety of nanoparticles.

### 2.13. Statistical analysis

All the data are presented as the mean ± standard deviation and were repeated at least three times. One-way analysis of variance (ANOVA) was used to assess the significance of the difference and SPSS version 22.0 (SPSS Inc., Chicago, IL) was used for statistical analysis. A *p* value <.05 was considered statistically significant.

## 3. Results and discussion

### 3.1. Synthesis and characterization of HA-Cur polymer

**Figure 1.**
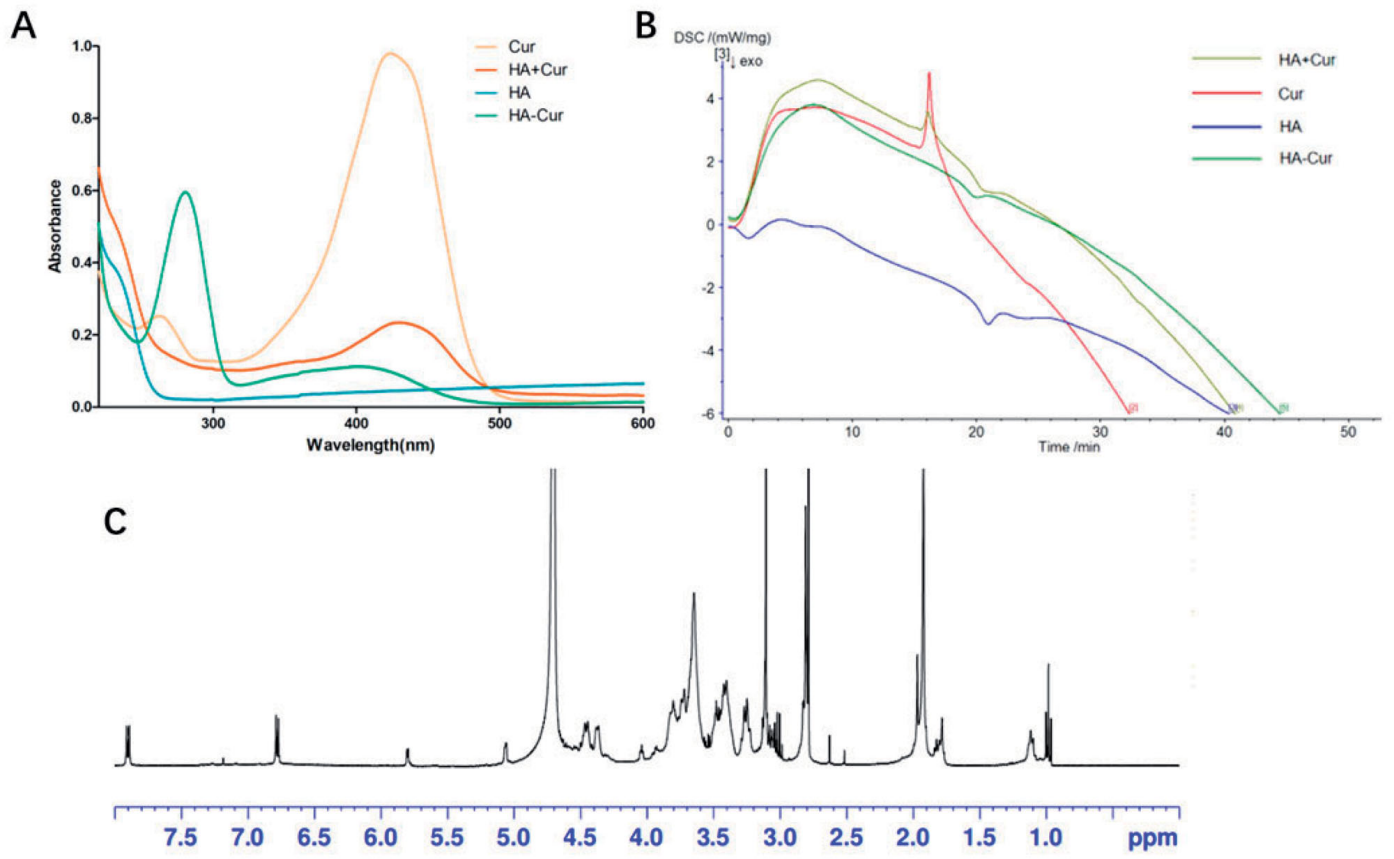
Structural confirmation of the HC complex. (A) Ultraviolet (UV) spectra of Cur, HA, HA + Cur, and HC. (B) DSC profile of Cur, HA, HA + Cur, and HC. (C) ^1^H NMR spectrum of the polymer HC.

In this study, the HA-Cur polymer was obtained by one-step esterification. The structure was confirmed by UV–visible spectrophotometer, DSC, and ^1^H NMR. The UV–vis spectra are shown in Figure 1(A). Compared with Cur and HA, HA + Cur had a wide absorption at 320–480 nm, which is similar to Cur. Meanwhile, HA-Cur also had a wide absorption at 320–480 nm, and a red shift at 290 nm, which may be caused by the introduction of the conjugated system. The conjugation of HA and Cur was evaluated using differential scanning calorimetry (Figure 1(B)). The curve of HA + Cur has both Cur and HA characteristic absorption peaks, and the green curve representing HA-Cur lacks the characteristic absorption peak of Cur, indicating that HA-Cur is not a simple physical mixture. In addition, in the ^1^H NMR spectra (Figure 1(C)), the peaks of HA-Cur at 1.9–2.0 ppm and 6.6–6.9 ppm belonged to HA (acetyl peak, −NHCOCH_3_) and CUR (aromatic protons), respectively. All the results indicated that Cur was successfully combined with HA, and the HA-Cur polymer was successfully prepared.

**Figure 2.**
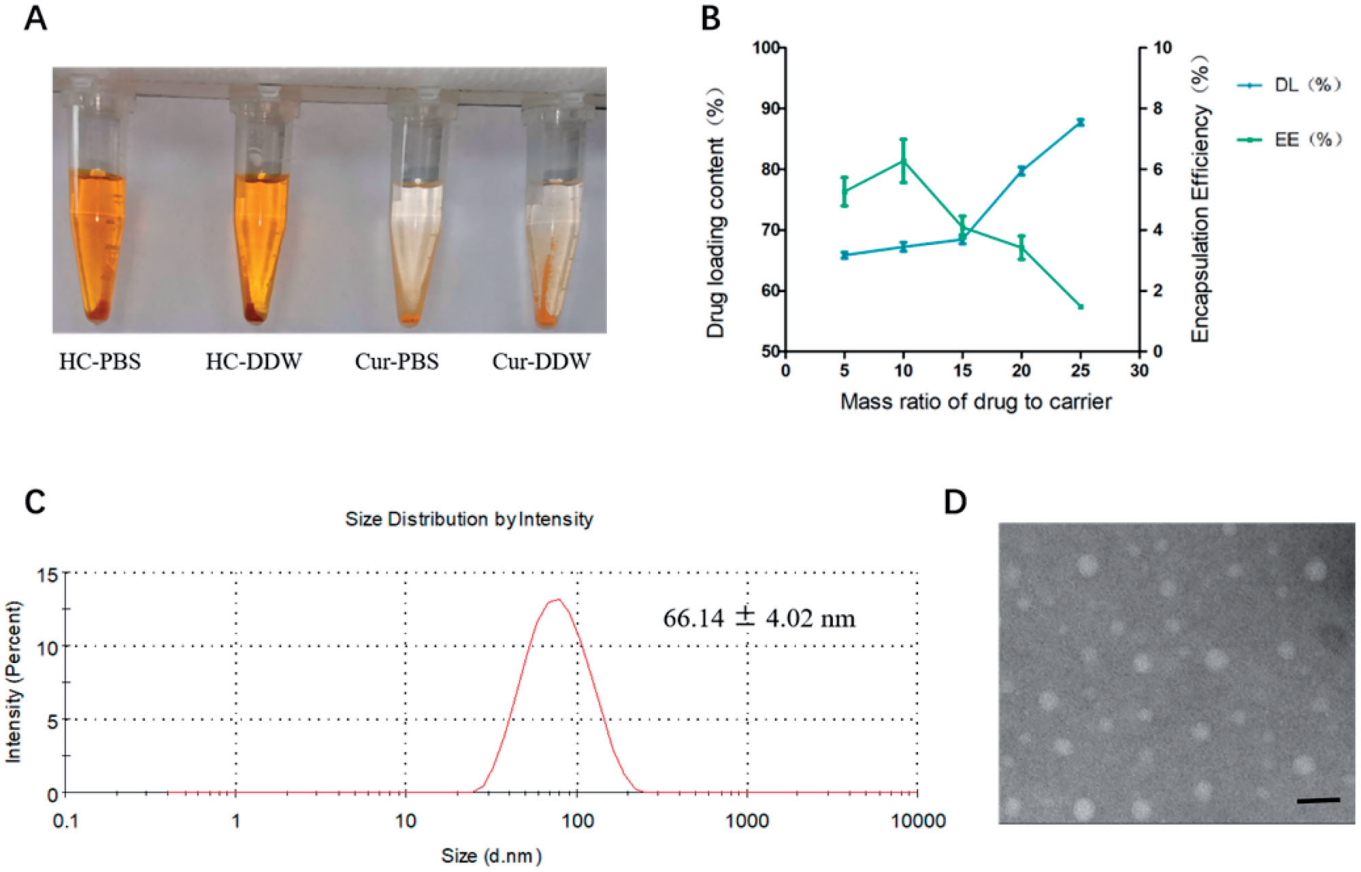
Optimal prescription screening. (A) Saturated aqueous solution and saturated PBS solution of HC and CUR. (B, C) Variation in the DAS encapsulation rate and drug loading under different dosing conditions. (D) The particle size of THCD-NPs under the optimal prescription. The data represented as the mean ± SD (*n* = 3). Scale bar = 100 nm.

During the preparation of the HC complex, the introduction of the hydrophilic substance HC could improve the water solubility of the hydrophobic substance Cur. The solubility test results of the two are shown in Figure 2(A). We also determined the solubility of CUR in each saturated solution by HPLC. The saturated solubility of free CUR in water and PBS buffer was 0.77 ± 0.01 μg/mL and 1.39 ± 0.05 μg/mL, respectively, while the solubility of the HC complex was 571.63 ± 6.41 μg/mL and 371.27 ± 3.28 μg/mL, respectively. It is obvious that the solubility of Cur in water and PBS solution is significantly improved after coupling Cur with HA.

### 3.2. Preparation and characterization of THCD-NPs

In this study, we first investigated the influence of the ratio of different carriers on micelles using the particle size of blank micelles, DAS DL and EE as evaluation indicators. As shown in Table 1, as the ratio of HC:TPGS decreased, the particle size of micelles gradually increased, but the DL of Cur decreased, when the ratio was 6:1, the PDI value was minimal. Therefore, HC:TPGS was selected as 6:1 as the final ratio.

**Table 1.** Carrier proportional screening.

HC/TPGS	Size (nm)	PDI	DL/Cur (%)
9:1	54.81 ± 0.72	0.27 ± 0.04	2.430 ± 0.003
6:1	58.93 ± 1.24	0.21 ± 0.01	2.314 ± 0.004
3:1	64.87 ± 2.13	0.23 ± 0.01	2.025 ± 0.002

The data represented as the mean ± SD (*n* = 3).

In addition, we examined the influence of the dosage of DAS on its DL and EE to determine the optimal dosage ratio. It can be seen from Figure 2(B) and Table 2, the EE of DAS gradually increased with the increasing proportion of DAS to carrier. When the ratio of DAS to carrier was 1:20, the DL of DAS reached the highest, but the encapsulation rate was low. After comprehensive consideration, 1:10 was selected as the final dosage ratio.

**Table 2.** Prescription screening.

DAS/carrier	DL/DAS (%)	EE/DAS (%)
1:25	3.45 ± 0.25	76.31 ± 4.06
1:20	3.69 ± 0.25	81.38 ± 6.15
1:15	3.17 ± 0.17	70.47 ± 3.14
1:10	5.94 ± 0.23	67.09 ± 3.33
1:5	7.54 ± 0.16	57.42 ± 0.54

The data represented as the mean ± SD (*n* = 3).

Based on the above results, we determined the best carrier ratio and the best dosage ratio. Subsequently, THCD-NPs were successfully prepared by the film dispersion method. The morphology, particle size, and PDI of THCD-NPs were analyzed by transmission electron microscopy and Malvern particle size analyzer. Then, after the micelles were hydrolyzed with strong alkali, the contents of Cur and DAS in THCD-NPs were determined by HPLC, and the DL and EE were calculated. As shown in Figure 2(C), the particle size of micelles was 66.14 ± 4.02 nm. The TEM results showed that THCD-NPs were uniformly spherical and well dispersed (Figure 2(D)). In addition, the DL of Cur was 2.20%±0.04%, and the DL and EE of DAS were 5.94%±0.23% and 67.09%±3.33%, respectively.

### 3.3. Characterization of the stability of THD-NPs

**Figure 3.**
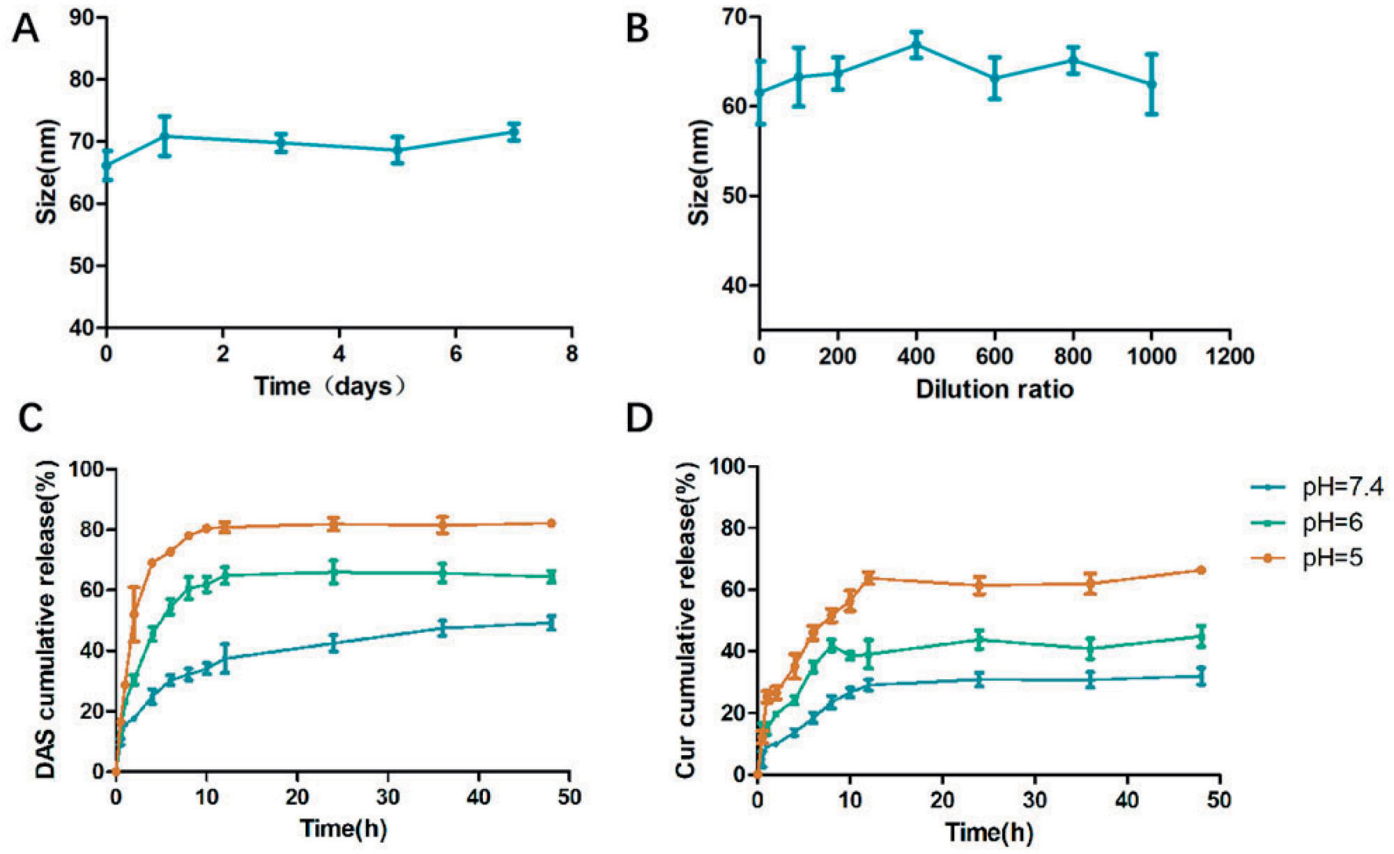
Investigation of *in vitro* stability and release. (A, B) *In vitro* stability and dilution stability of THCD-NPs. Drug release tendencies of DAS (C) and Cur (D) are demonstrated graphically. The data represented as the mean ± SD (*n* = 3).

The stable particle size of micelles can be conducive to drug self-delivery by improving the biological distribution of drugs. Therefore, it is necessary to investigate the stability of THCD NPs under physiological conditions. We stored THCD-NPs in a PBS solution containing 10% fetal calf serum for one week, and their hydrated particle size did not change significantly, indicating their good stability (Figure 3(A)).

In addition, because the *in vivo* treatment is administered intravenously, the micellar solution will be diluted in the blood and cause its disintegration, so we investigated the dilution stability of THCD-NPs. Figure 3(B) shows that the particle size of the mixed micelles remained at approximately 60 nm when they were diluted 1000 times, indicating that THCD-NPs have good anti-dilution ability *in vivo* and can be used for intravenous administration.

### 3.4. The vitro drug release of THCD-NPs

The effective release of the drugs in tumor sites was expected by us. In this study, we constructed a carrier with acid-sensitive ester bonds to respond to the acidic environment of the lysosome in tumor cells or the tumor microenvironment. PBS solutions with different pH values (pH 7.4, 6.0, and 5.0) were used to simulate the normal physiological environment, tumor microenvironment, and lysosome internal environment to study the release of Cur and DAS from THCD-NPs under different pH conditions.

Figure 3(C,D) shows the release curves of THCD NPs under different pH conditions. At pH 7.4, the release of Cur and DAS was slow and the release amount was small. After incubation for 48 h, only approximately 30% of Cur and 49% of DAS were released from THCD-NPs. In contrast, when the pH values decreased to 6.0 and 5.0, the release rates of Cur and DAS increased significantly. The release of Cur after 48 h of incubation was approximately 44% (pH 6.0) and 66% (pH 5.0), and that of DAS after 48 h of incubation was about 64% (pH 6.0) and 82% (pH 5.0). These results indicated that THCD-NPs remained relatively stable in the physiological environment and successfully achieved pH-responsive drug release, which helps to improve the therapeutic effect of the drug and reduce the risk of potential adverse reactions.

### 3.5. Cellular uptake of THCD-NPs

**Figure 4.**
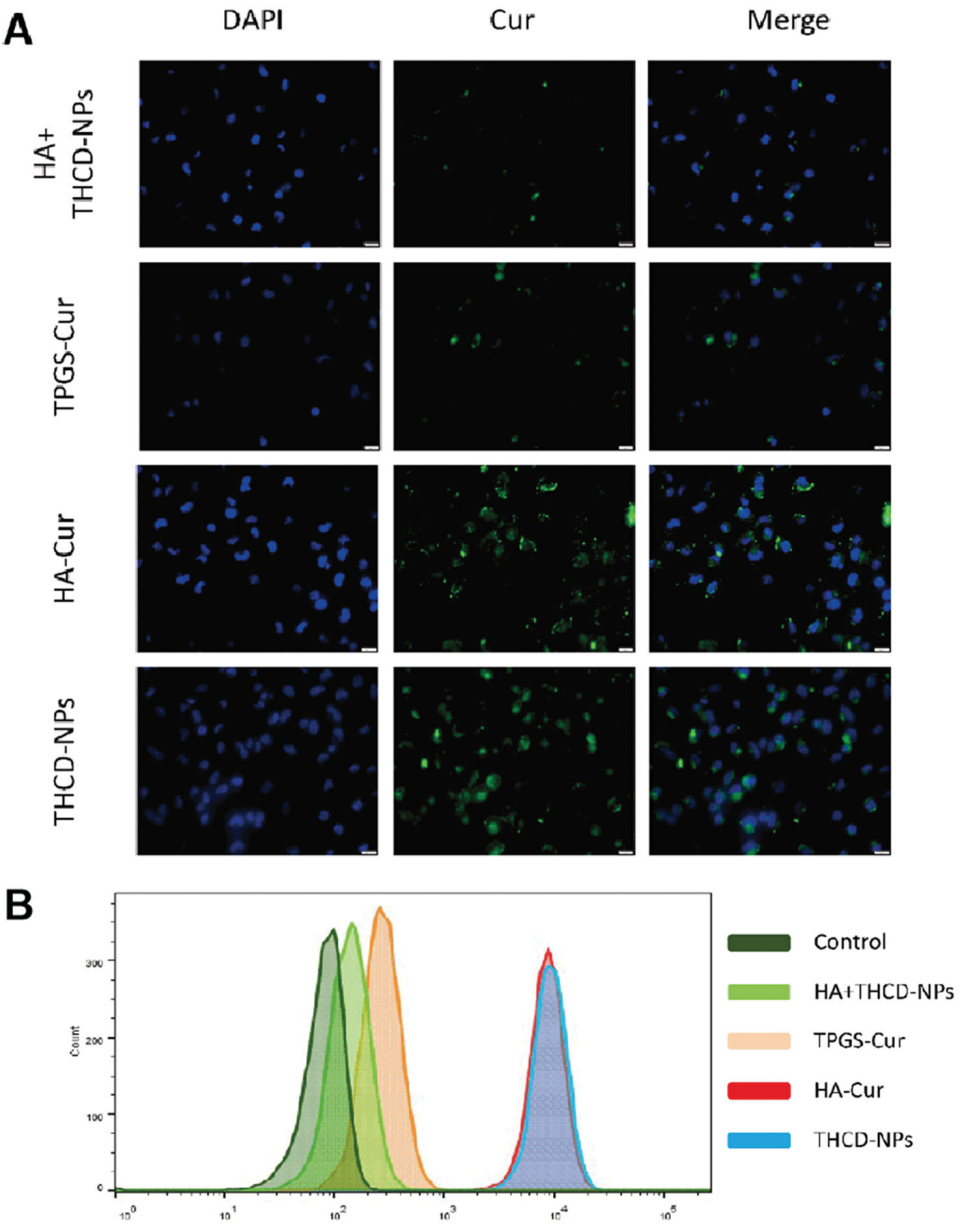
Cellular uptake of drugs. Fluorescent micrographs (A) and flow cytometry results (B) depicting the cellular accumulation of HA + THCD-NPs, TPGS-Cur, HA-Cur, and THCD-NPs after 4 hours of co-incubation with HepG2 cells. Scale bar = 20 μm.

To determine whether the introduction of HA can promote the internalization of THCD-NPs, the uptake of THCD-NPs by HepG2 cells was quantitatively observed using Cur as a fluorescent marker. Figure 4(A) shows that after four hours of incubation, the fluorescence signal of Cur in the THCD-NPs and HA-Cur treated cells was stronger than that in the HA pre-incubation and TPGS-Cur group. As the pre-incubated free HA bound to the CD44 protein, it hindered the binding of THCD-NPs to the CD44 protein, and the nanoparticles lost their active targeting ability. Meanwhile, due to the lack of active targeting ability of nanoparticles encapsulated by TPGS, the intracellular fluorescence intensity of TPGS-Cur treated group was lower. Therefore, there was almost no Cur fluorescence in HA + THCD-NPs and TPGS-Cur treated cells, and a small amount of fluorescence may be caused by passively targeted endocytosis.

Additionally, corresponding flow cytometry studies confirmed this observation, showing significantly enhanced Cur fluorescence in the THCD-NPs and HA-DAS groups with HA active targeting (Figure 4(B)), while decreased Cur fluorescence in the HA pre-incubation group and TPGS-Cur group (Figure 4(B)). These results suggest that the introduction of HA into the micellar system can effectively promote the targeting of THCD-NPs to tumor cells.

### 3.6. Cytotoxicity of THCD-NPs *in vitro*

**Figure 5.**
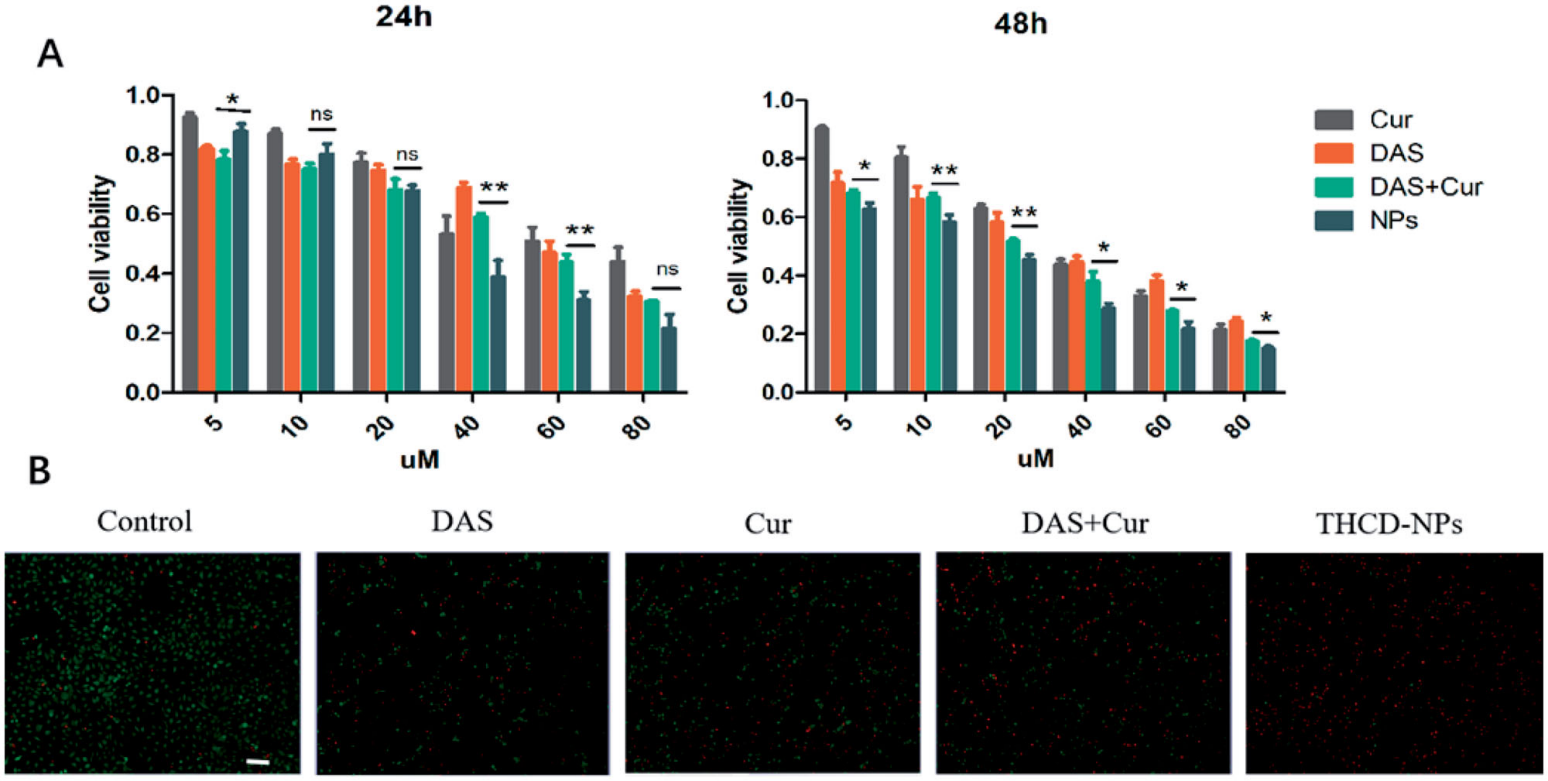
Evaluation of *in vitro* anti-cancer effect of THCD-NPs. (A) Graphical representations of the dose-dependent cytotoxic effects of free drugs and THCD-NPs on HepG2 cells separately, as depicted from the MTT assay conducted after 24 and 48 hours of treatment. (B) Fluorescent micrographs depicting the cytotoxicity of different drugs. Green represents living cells and red represents dead cells. *n* = 3, *p<.05, **p<.01 (DAS + Cur vs. THCD-NPs). Scale bar = 200 μm.

In previous studies, we demonstrated that THCD-NPs can effectively release drugs in the tumor microenvironment and can be effectively taken up by tumor cells. To further understand the cytotoxicity of THCD-NPs, HepG2 cells were incubated with different concentrations of Cur, DAS, Cur + DAS, and THCD-NPs for 24 h or 48 h. As shown in Figure 5(A), after incubation for 24 h or 48 h, compared with free Cur and DAS, Cur + DAS could effectively increase the killing effect of HepG2 cells, which may be due to the synergistic effect of Cur and DAS in blocking different cell cycles. In addition, the cytotoxicity of THCD-NPs on HepG2 cells was significantly stronger than that of the combined use of the two drugs (*p*<.05), which may be related to the increased water solubility of the two drugs and the active targeting ability mediated by nanoparticles. Meanwhile, with increasing incubation time and drug concentration, the cell survival rate also decreased, indicating that the cytotoxicity of the preparation was concentration-dependent and time-dependent. The IC_50_ value also reflected this result (Table 3).

**Table 3.** IC_50_ values for each treatment group.

Groups	IC_50_ (μM)	
	24 h	48 h
DAS	58.30 ± 3.27	24.40 ± 3.89
Cur	56.30 ± 7.84	29.48 ± 3.21
DAS + Cur	44.24 ± 5.92*	18.02 ± 1.74**
THCD-NPs	30.13 ± 5.71	12.55 ± 0.60

*n* = 3.

* *p*<.05.

** *p*<.01 (DAS + Cur vs. THCD-NPs).

Furthermore, HepG2 cells were incubated with 40 μM Cur, DAS, Cur + DAS, and THCD-NPs for 24 h, and then the viability of the HepG2 cells was measured by co-staining with calcine AM and PI to differentiate live (green) and dead (red) cells (Figure 5(B)). These results are consistent with the MTT results and show that the THCD-NPs have a remarkable cell-killing ability, making them a promising platform for cancer therapy.

### 3.7. Analysis of apoptosis and cell cycle

**Figure 6.**
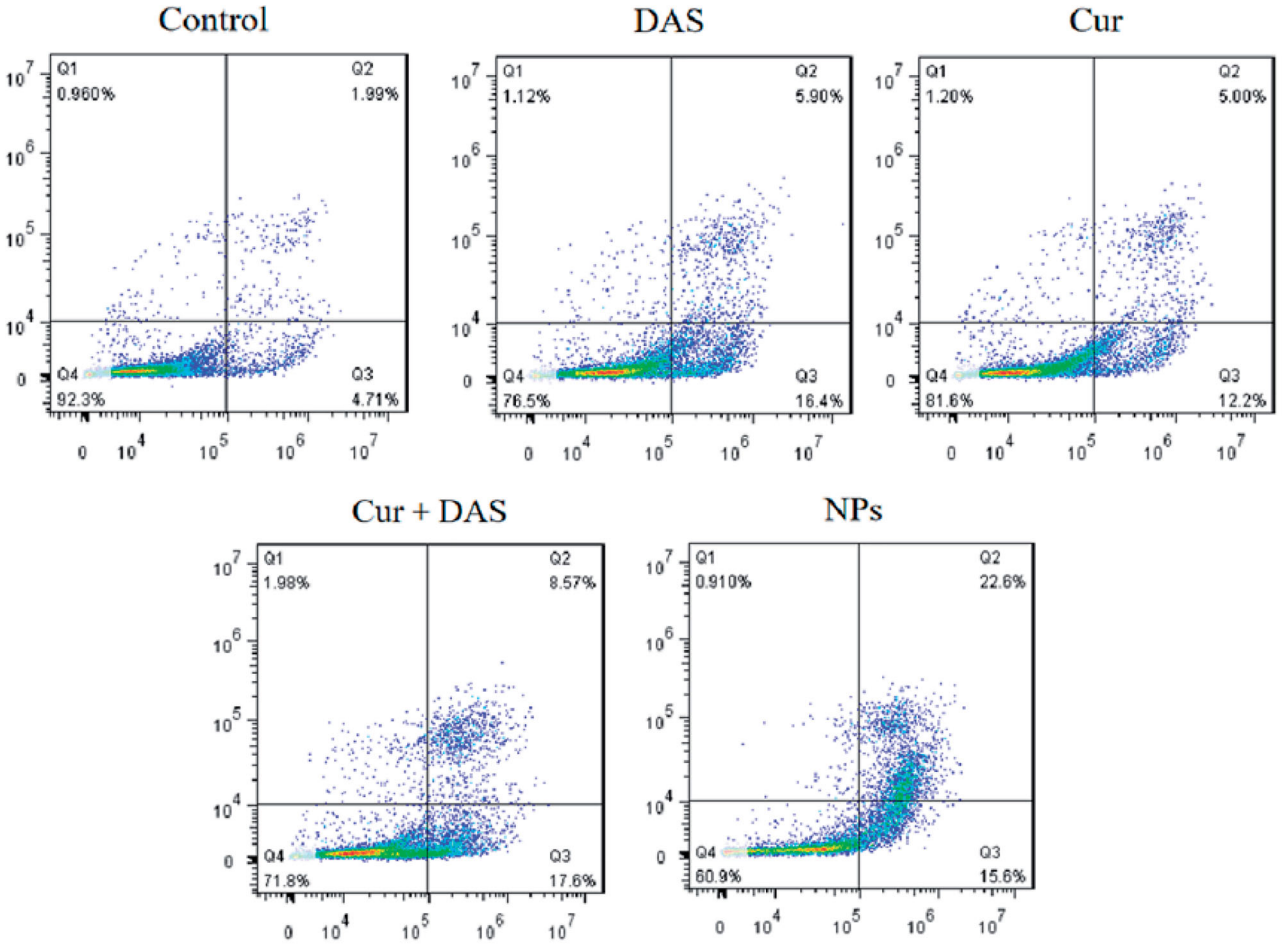
Quantitative investigation of cytotoxicity *in vitro*. After co-incubation of HepG2 cells with DAS, Cur, DAS + Cur, and THCD-NPs for 24 h, the apoptosis of each group was measured by flow cytometry.

**Figure 7.**
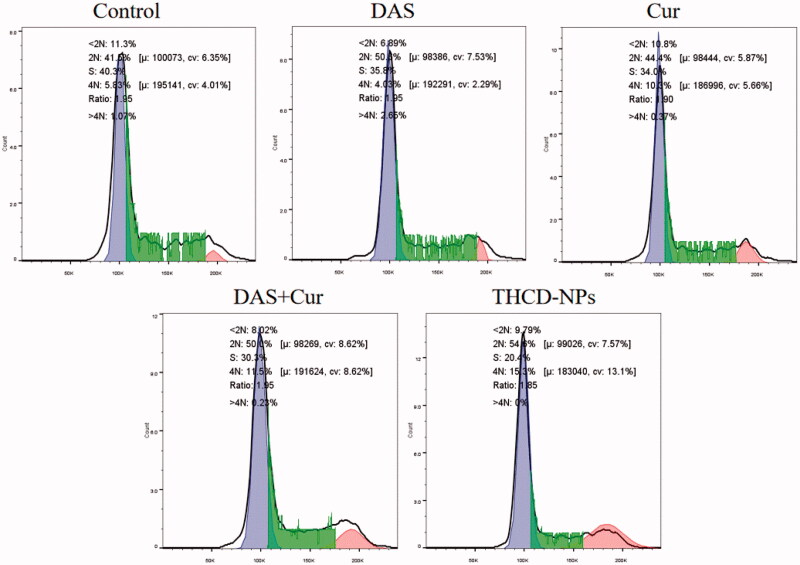
Cell cycle detection. After co-incubation of HepG2 cells with different drugs for 24 h, the cell cycle of each group was measured by flow cytometry.

To further verify the potential of THCD-NPs to inhibit HepG2 cells, cell apoptosis was detected by using the annexin V-FITC detection kit. In Figure 6, *Q*_1_, *Q*_2_, *Q*_3_, and *Q*_4_ represent living cells, early-apoptotic cells, late-apoptotic cells, and necrotic cells, respectively, and the apoptosis rate was the sum of *Q*_2_ and *Q*_3_. The results showed that the apoptosis rate increased from 6.7% in the control group to 38.2% in the THCD-NPs group. The apoptosis rate of the THCD-NPs group was approximately 1.46 times that of the Cur + DAS group. As shown in Figure 7, the proportion of living cells decreased from 81.6% and 76.5% in the Cur and DAS groups to 71.8% in the Cur + DAS group, and the proportion of apoptotic cells increased slightly from 17.2% and 21.4% to 26.17%. The results showed that the combination of Cur and DAS had a synergistic effect to promote cell apoptosis, and the apoptosis rate increased after the two drugs were made into nano micelles, which may be related to the increase in water solubility and targeting of the drugs.

To further investigate the cell death mechanism of nanomedicine, flow cytometry was used for cell cycle analysis. As shown in Figure 7, DAS inhibited HepG2 cell proliferation by inducing G1/S blockade. The apoptotic mechanism of HepG2 cells induced by Cur was mediated by inhibiting the G2/M cell cycle. The results were consistent with previous reports. When the two drugs were used in combination, they blocked the G1/S and G2/M cycles of HepG2 cells and exerted a synergistic effect. Meanwhile, the cycle arrest rate was increased in the THCD-NPs group, which was consistent with the possibility that HA-modified nanoparticles could target Cur and DAS to CD44 protein-positive cancer cells.

### 3.8. Hemolysis test

**Figure 8.**
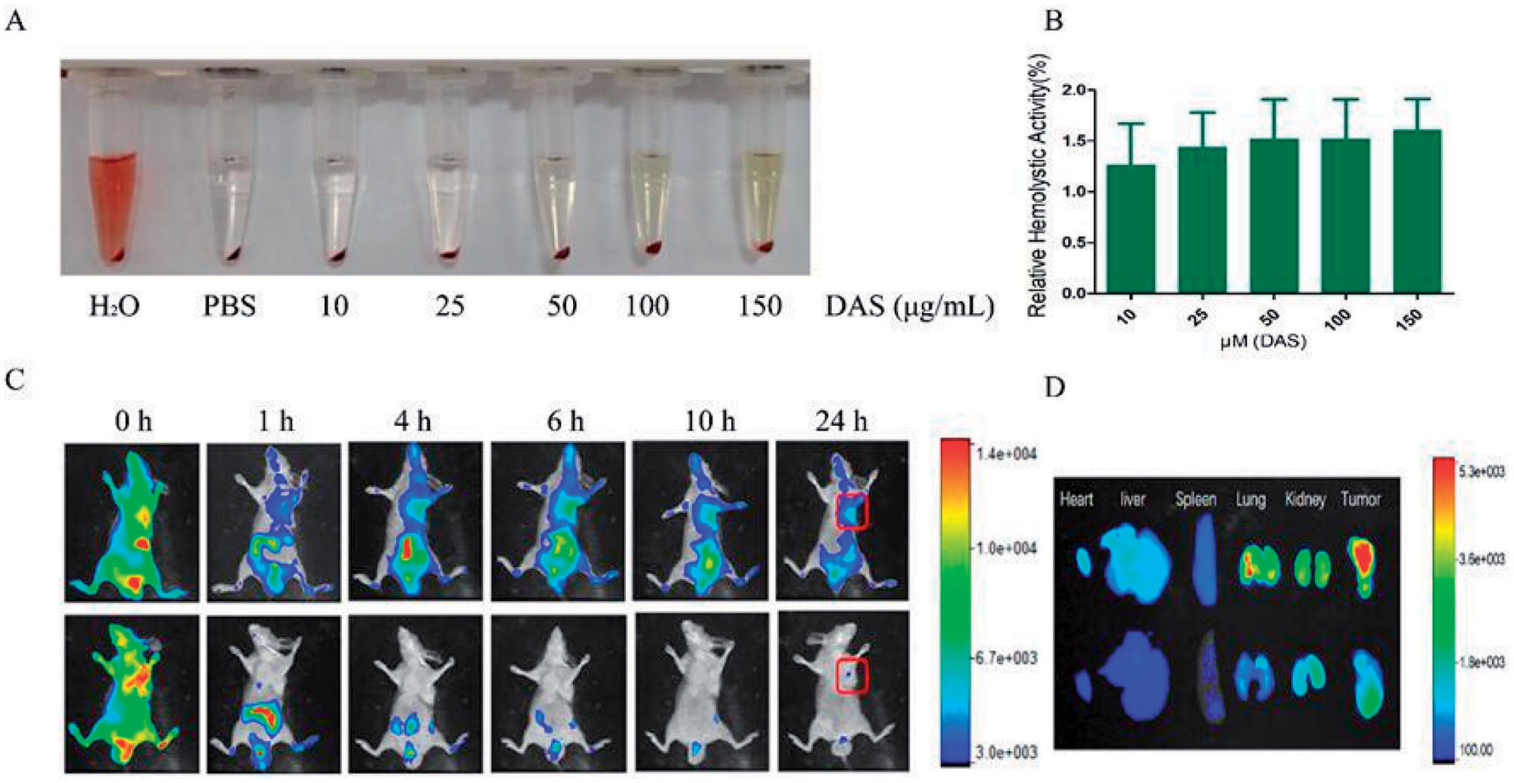
Fluorescence imaging results and hemolysis test examination results. Determination of hemolysis (A) and hemolysis rate (B) under different drug concentrations. *In vivo* fluorescence imaging of HepG2 tumors at different time points after intravenous injection (C) and (D) *ex vivo* fluorescence imaging of HepG2 tumors at 24 h after intravenous injection (the upper panel shows Cy5-THC-NPs, and the lower panel shows Cy5). The data represented as the mean ± SD (*n* = 3).

To evaluate the biosafety of THCD-NPs, the hemolysis of micelles was further studied. As shown in Figure 8(A,B), THCD-NPs showed no obvious hemolysis at different concentrations. Even when the DAS concentration reached 150 μg/mL, the relative hemolysis rate was still lower than 2%, indicating that micelles had a low impact on erythrocytes and could be used for injection.

### 3.9. *In vivo* distribution of THCD-NPs

To further research the targeting effect of THCD-NPs *in vivo*, we injected free Cy5 and Cy5-labeled nanoparticles into tumor-bearing nude mice through the tail vein. The results measured by fluorescence imaging technology at 0 h, 1 h, 4 h, 6 h, 10 h, and 24 h after injection are shown in Figure 8(C). The fluorescence brightness of the nanomicelle group was retained for a long time *in vivo* and was obviously accumulated at the tumor site. Twenty-four hours later, the heart, liver, spleen, lung, kidney, and tumor tissues of nude mice were collected and analyzed with a small animal imaging system. The results are shown in Figure 8(D). The fluorescence intensity of the nanomicelle group at the tumor site was significantly higher than that of the Cy5 free group, indicating that the nanomicelles had good tumor tissue targeting and aggregation.

### 3.10. Antitumor effects of THCD-NPs

**Figure 9.**
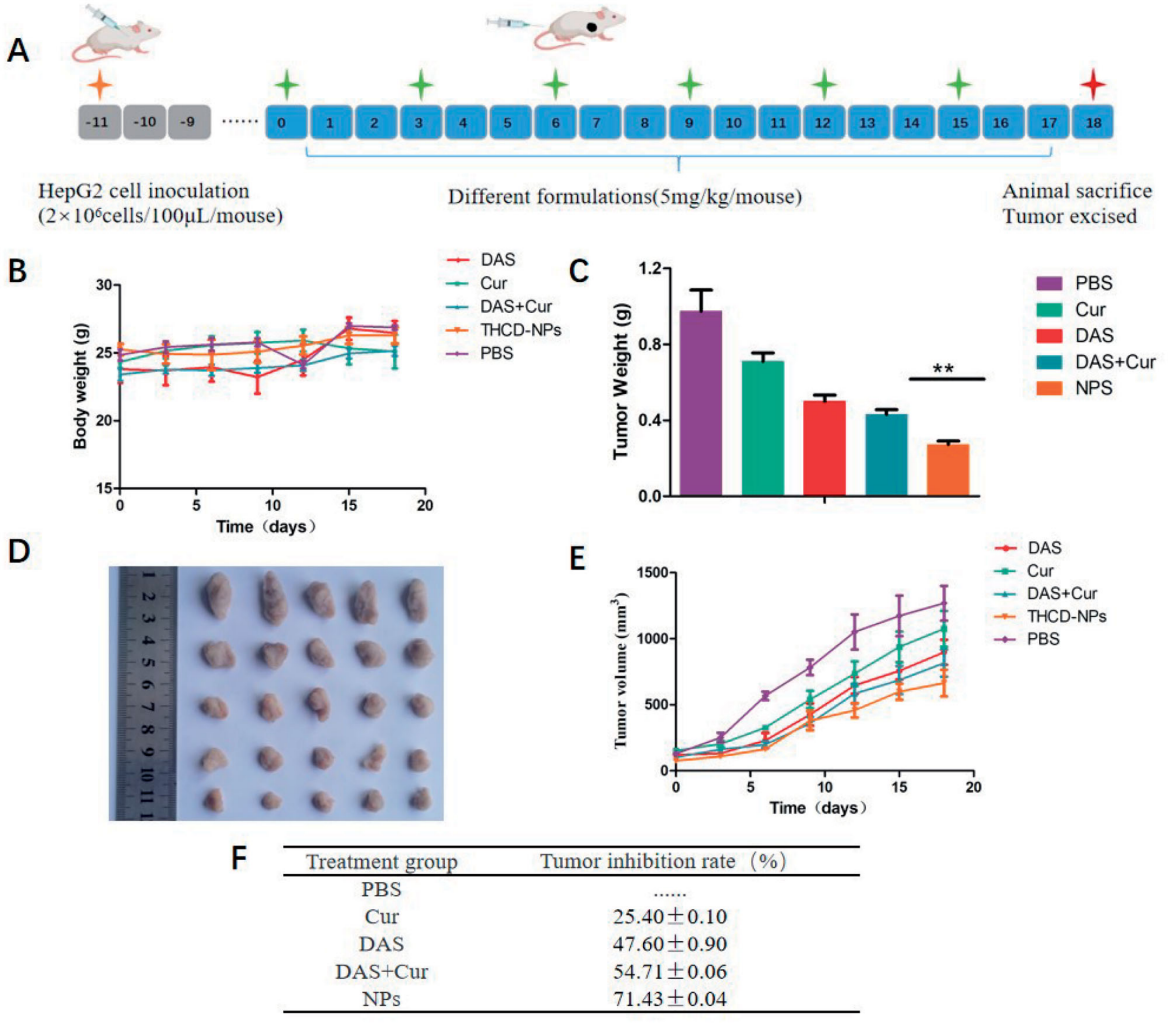
Evaluation of the anti-tumor effect of THCD-NPs *in vivo*. (A) The experimental process. Body weight changes (B) and tumor volume changes (E) of mice in each group during the treatment period. Images of isolated tumors (D) and tumor weight (C) in each group after the end of treatment. (F) The relative tumor inhibition rate of each treatment group. The data represented as the mean ± SD (*n* = 5). **p<.01 (DAS + Cur vs. THCD-NPs).

The experimental results of the antitumor effects of nanoparticles *in vitro* were encouraging, so it was necessary to further evaluate their antitumor effects *in vivo*. As shown in Figure 9(A), the transplanted tumor model was established by subcutaneous injection of HepG2 cells. The mice were randomly divided into five groups (*n* = 5), and the drug was administered once every three days six times when the tumor volume reached approximately 100 mm^3^. The body weight and tumor volume of the mice were recorded before each administration, and the treatment ended on the 18th day. As shown in Figure 9(C–F), the effects of PBS on tumor growth were negligible, while HepG2 tumor growth was significantly inhibited in the Cur + DAS and THCD-NPs groups, and the therapeutic effect of the THCD-NPs group was superior to that of the Cur + DAS group.

Notably, there was no significant fluctuation in body weight in each group during administration (Figure 9(B)), which indicates that micellar nanoparticles have better safety. Furthermore, we calculated the relative tumor inhibition rate (TGI) of each treatment group, and the results are shown in Figure 9(F). The TGI of the THCD-NPs treatment group was 71.43 ± 0.04%, which was higher than that of the DAS + Cur treatment group (54.71 ± 0.06%).

**Figure 10.**
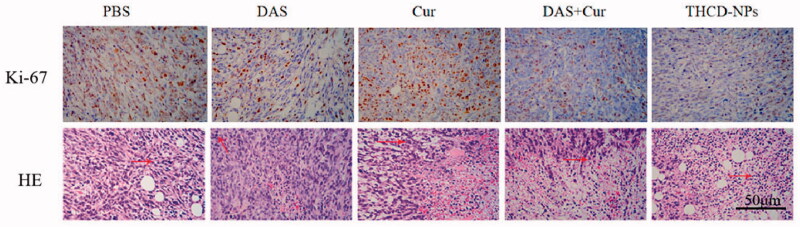
HE staining results and immunohistochemical results of isolated tumors in each group after the end of treatment. Scale bar = 50 μm.

We further studied the potential anti-tumor mechanism of THCD-NPs, and analyzed tumor tissues by HE staining. In the THCD-NPs treated group, as shown by the arrow in Figure 10 (the upper panel), large area of apoptosis occurred in tumor tissues. Ki67 is a kind of cell proliferation and antigen that indicates cell proliferation. The measurement results are shown in Figure 10 (the lower panel). Compared with the other groups, the Ki67 expression signal quantity was the weakest in the THCD-NPs treatment group, indicating that tumor cell proliferation was significantly reduced and apoptotic cells increased after treatment. Based on the above results, the efficacy of the two drugs *in vivo* after the preparation of nano-micelles was better than the combined application of free drugs, which was related to the improvement of water solubility and targeting of the two drugs by micelles.

### 3.11. *In vivo* biological safety

**Figure 11.**
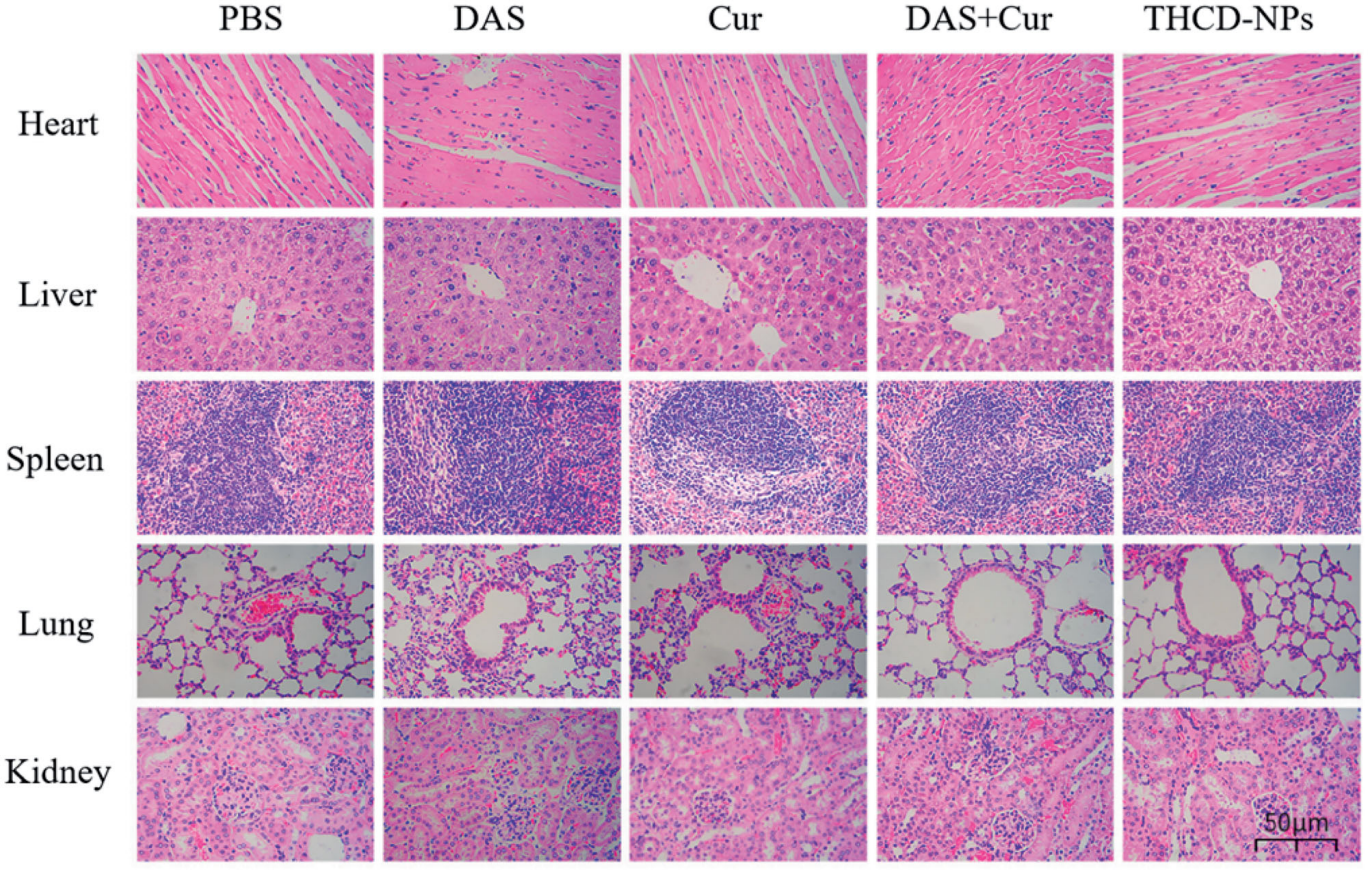
HE staining results of organs in each treatment group. Scale bar = 50 μm.

**Figure 12.**
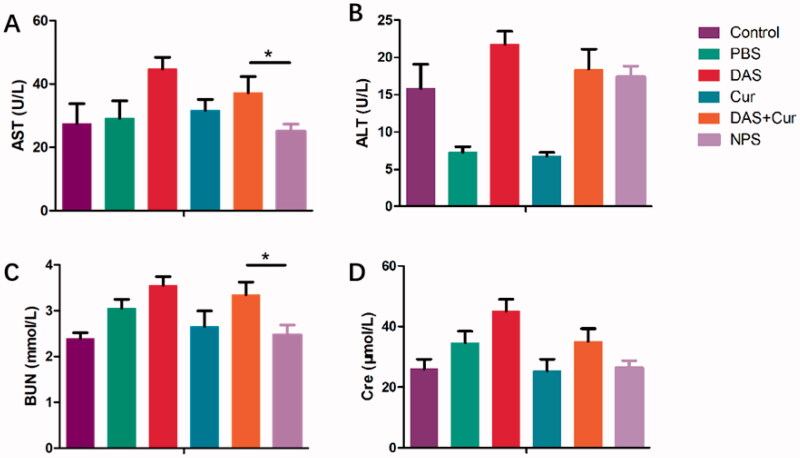
Evaluation of liver and kidney functions of mice in each treatment group. The data represented as the mean ± SD (*n* = 5). *p<.05 (DAS + Cur vs. THCD-NPs).

The biosafety of the THCD-NPs system *in vivo* was further investigated after its superior antitumor activity was confirmed. Morphological and histological changes in major tissues were examined using HE staining to further assess the biosafety of the THCD-NPs system. As shown by the red arrow in Figure 11, there were no obvious pathological changes in any group of organs. The THCD-NPs prepared in this study has good safety. Moreover, serum AST and ALT were used to evaluate liver function (Figure 12(A,B)). Blood urea nitrogen and Cre were used to assess renal function (Figure 12(C,D)). Compared with the normal group, several indexes of the DAS treatment group increased significantly, but there was no significant difference between the THCD-NPs treatment group and the normal group. This was consistent with the HE results.

## 4. Conclusions

In summary, we successfully designed a pH-responsive and CD44 receptor-targeted nanocervator (HA-Cur) and then characterized it by ^1^H NMR. Micelles containing DAS with smaller particle size, better DL, and higher EE (THCD-NPs) were obtained by self-assembly. THCD-NPs had good stability and could continuously release DAS and Cur under weakly acidic conditions. In addition, the co-delivery of Cur and DAS can be realized by using HA-cur as the vector and can effectively target CD44 overexpressed HepG2 cells. The combined use of Cur and DAS to block tumor cells in different cycles resulted in a synergistic effect. In conclusion, this finding not only provides a new perspective for the preparation of high-performance nanomicelles based on oligomeric HA but also offers strong evidence for the treatment of liver cancer by CD44 targeting and pH-sensitive drug delivery strategies.
